# Protective effects of sodium butyrate on rotavirus inducing endoplasmic reticulum stress-mediated apoptosis via PERK-eIF2α signaling pathway in IPEC-J2 cells

**DOI:** 10.1186/s40104-021-00592-0

**Published:** 2021-06-11

**Authors:** Ye Zhao, Ningming Hu, Qin Jiang, Li Zhu, Ming Zhang, Jun Jiang, Manyi Xiong, Mingxian Yang, Jiandong Yang, Linyuan Shen, Shunhua Zhang, Lili Niu, Lei Chen, Daiwen Chen

**Affiliations:** 1 College of Animal Science and Technology, Sichuan Agricultural University, Huimin Road 211#, Chengdu, Sichuan Province 611130 P. R. China; 2 Institute of Animal Nutrition, Sichuan Agricultural University, Huimin Road 211#, Chengdu, Sichuan Province 611130 P. R. China; 3 Key Laboratory for Animal Disease-Resistance Nutrition of China Ministry of Education, Sichuan Agricultural University, Chengdu, Sichuan 611130 P. R. China

**Keywords:** Apoptosis, IPEC-J2, PERK-eIF2α, Rotavirus, Sodium butyrate

## Abstract

**Background:**

Rotavirus (RV) is a major pathogen that causes severe gastroenteritis in infants and young animals. Endoplasmic reticulum (ER) stress and subsequent apoptosis play pivotal role in virus infection. However, the protective mechanisms of intestinal damage caused by RV are poorly defined, especially the molecular pathways related to enterocytes apoptosis. Thus, the aim of this study was to investigate the protective effect and mechanism of sodium butyrate (SB) on RV-induced apoptosis of IPEC-J2 cells.

**Results:**

The RV infection led to significant cell apoptosis, increased the expression levels of ER stress (ERS) markers, phosphorylated protein kinase-like ER kinase (*PERK*), eukaryotic initiation factor 2 alpha (*eIF2α*), caspase9, and caspase3. Blocking PERK pathway using specific inhibitor GSK subsequently reversed RV-induced cell apoptosis. The SB treatment significantly inhibited RV-induced ERS by decreasing the expression of glucose regulated protein 78 (*GRP78*), *PERK*, and *eIF2α*. In addition, SB treatment restrained the ERS-mediated apoptotic pathway, as indicated by downregulation of C/EBP homologous protein (*CHOP*) mRNA level, as well as decreased cleaved caspase9 and caspase3 protein levels. Furthermore, siRNA-induced GPR109a knockdown significantly suppressed the protective effect of SB on RV-induced cell apoptosis.

**Conclusions:**

These results indicate that SB exerts protective effects against RV-induced cell apoptosis through inhibiting ERS mediated apoptosis by regulating PERK-eIF2α signaling pathway via GPR109a, which provide new ideas for the prevention and control of RV.

## Introduction

Rotavirus (RV) is the main cause of viral gastroenteritis in infants, young children, and young animals around the globe [1–3], which RV infection results in 12,000 to 15,000 annual deaths among children under 5 years each year [4, 5]. In addition, RV is responsible for 7%–50% mortality in piglets, causing great economic losses to the pork industry [6, 7]. The RV is transmitted via the faecal-oral route. The faeces from an infected host contain more than 10 trillion pfu/gr of viruses, but less than 100 of them can transmit infection and make someone else sick [8]. The RV primary infects mature enterocytes and results in blunting, atrophy and fusion of villi, denudation of tip of villi and cryptal cells hyperplasia, thereby disrupting their physiological and absorptive function, which lead to diarrhoea [9, 10]. Epidemiological researches have confirmed that RV in environment could contribute to the development of infectious gastrointestinal illness, which raising serious concerns about impacts on public health [11, 12]. The detrimental effects of RV on public health have prompted substantial concern about how to efficiently protect the human or animal against RV infection.

Cell perception of various extracellular stimuli, such as viral infection, triggers specific intracellular signaling networks that results in cell apoptosis [13–15]. The RV infection causes intestinal barrier dysfunction and disrupts intestinal homeostasis, which could induce the apoptosis of epithelial cells [9, 16]. Endoplasmic reticulum (ER) is a vital organelle that performs a variety of intracellular processes, including synthesis, folding, and post-translational modifications of proteins, and apoptosis, whose homeostasis is crucial for epithelial cells [17, 18]. Emerging reports have confirmed that virus infection could impair ER homeostasis in host cells and eventually lead to ER stress (ERS) [17, 19]. The protein kinase RNA-like ER kinase (*PERK*) is one of the major ER transmembrane protein that is phosphorylated upon ERS. Subsequently, activation of *PERK* lead to the phosphorylation of α-subunit of eukaryotic initiation factor 2 alpha (*eIF2α*) [20]. Phosphorylated *eIF2α* promotes the induction of activating transcription factor 4 (*ATF4*), which induces the expression of pro-apoptotic C/EBP homologous protein (*CHOP*) [21]. The *CHOP* promoted apoptosis by increasing expression of the pro-apoptotic factor Bax and suppressing the expression of the anti-apoptotic Bcl-2 [22, 23]. So far, numerous viruses have been demonstrated to induce cell apoptosis via PERK-eIF2α-CHOP signaling pathway in infected cells [24, 25]. Kaposi’s sarcoma-associated herpesvirus induces ERS, caspase activation, and *CHOP* expression, which in turn results in apoptosis of primary effusion lymphoma cells [25]. Venezuelan equine encephalitis virus induced apoptosis through PERK-eIF2α-CHOP signaling pathway in infected U87MG cells [26]. Japanese encephalitis virus infection induced cell apoptosis by activating the PERK-ATF4-CHOP pathway *in vitro* and *in vivo* [27]. Nevertheless, whether RV could cause ERS-mediated cell apoptosis remains to be investigated.

Sodium butyrate (SB), a salt form of four-carbon short-chain fatty acid, has been endogenously produced by bacterial fermentation of dietary fibers in the colon [28]. Published studies have reported that SB has a wide range of pharmacological properties, including anti-inflammation, anti-oxidation, antitumor activities, and metabolism regulation [29–33]. The SB protects against lipopolysaccharide-induced endometritis through inhibiting inflammatory response in mice [34]. The latest study showed SB prevented tert-butyl hydroperoxide-induced oxidative stress and apoptosis in human nucleus pulposus cells [35]. The SB also could protect islet cells from apoptosis through inhibiting the PERK-CHOP pathway of ERS [36]. Despite, those results suggested that SB might play a critical role in reducing cell apoptosis. It is unknown whether SB could prevent RV-induced cell apoptosis. Therefore, based on the established RV infected intestinal epithelial cell model [37], we investigate for the first time the protective effect and mechanism of SB against RV-induced apoptosis via the PERK-eIF2α signaling pathway in IPEC-J2 cells. These findings provide new ideas for prevention and control of RV.

## Materials and methods

### Cell culture and viral infection

The IPEC-J2 cell line (obtained from professor Per Torp Sangild, University of Copenhagen, Denmark) is isolated from the mid-jejunum epithelium of a neonatal un-suckled piglet. The IPEC-J2 cells were planted in DMEM/F12 medium supplemented with 10% fetal bovine serum, antibiotics (1% penicillin-streptomycin), 5 mg/mL hEGF, and 10 nmol/L HEPES, under an incubator of 5% CO_2_ at 37 °C. The RV strain was purchased from China Institute of Veterinary Drug Control. Confluent (80%) IPEC-J2 cells were infected with RV at multiplicity of infection (MOI) of 10 at 37 °C for 1 h. After that, the inoculum was carefully removed, and the cells were washed twice with PBS and cultured in fresh growth medium.

### PERK inhibitor treatments

The cells were pre-treated with the *PERK* inhibitor GSK (1.0 μmol/L and 10 μmol/L) for 24 h, followed by challenge with RV (10 MOI) for 1 h, and then cultured with DMEM/F12 for a further 24 h.

### SB treatments

The SB (Sigma Aldrich, St. Louis, MO, USA) was dissolved in DMEM/F12 medium. Experimental procedures were based on the methods in our laboratory [37]. The concentration of SB was selected based on previous studies [38, 39]. Briefly, cells were cultured with different concentrations of SB (0, 1, 2, 4, 8, and 16 mmol/L) at 37 °C with 5% CO_2_ for 24 h, followed by removing medium and washing with PBS three times, then challenged with RV at MOI of 10 for 1 h. Next, removal of the inoculums and washing twice with PBS, the cells were incubated with basal medium (serum free) containing SB (0, 1, 2, 4, 8, and 16 mmol/L) for a further 24 h.

### RNA interference

GPR109a-specific siRNA1 (CGATGTTAATCAAGAAGCA), siRNA2 (GTAGCTTCAGCAT CTGCAA), and negative control siRNA (siCtrl) (RiboBio, Guangzhou, china) were used to knockdown GPR109a. The si-GPR109a and siCtrl were transfected into IPEC-J2 using lipofectamine 3000 (Invitrogen, USA) following the manufacturer’s procedures.

### Determination of cell viability and apoptosis

The IPEC-J2 cells under different conditions seeded in sterile 96-well plates with cell density of 4 × 10^4^/mL with 100 μL medium. The Cell Counting Kit-8 (CCK-8) kit (Dojindo, Kumamoto, Japan) was adopted to measure the cell viability. Cell apoptosis was detected by Fluorescein isothiocynate (FITC)-, Alexa Fluor®647- conjugated Annexin V with propidium iodide (PI) staining assay (Biolegend) according to the manufacturer’s protocols. Briefly, IPEC-J2 cells from the control and RV (GSK or SB or siRNA1) -treated groups were harvested and rinsed twice with PBS. Then, cells were resuspended in 100 μL 1 × binding buffer and incubated with Alexa Fluor®647- conjugated Annexin V (2 μL/10^6^ cells) for 20 min on ice. Subsequently, 400 μL 1 × binding buffer and 1 μL PI (1 mg/mL) were added successively and immediately analyzed by flow cytometry.

### Real-time quantitative PCR

Total RNA was isolated from IPEC-J2 cells using RNAiso reagent (Invitrogen, Carlsbad, CA, USA) according to the manufacturer’s instructions, followed by the synthesis of cDNA by the prime script™ RT reagent kit with gDNA eraser (Takara, Dalian, China). The RNA purity and integrity were assessed by spectrophotometric (A260 and 280 nm ratio) analysis and agarose gel (1%) electrophoresis, respectively. Real-time quantitative PCR was carried out with a SYBR Premix EX Taq kit (TaKaRa, Dalian, China) and the CFX96 Real-Time PCR Detection System (Bio-Rad, Hercules, CA, USA). Relative gene expression was calculated with the 2^−ΔΔCT^ method, normalizing the results to the value for the *β-actin* gene. Primer sequences used in this experiment are shown in Table 1.

**Table 1:** Primer sequences and optimal annealing temperatures (OAT) of genes selected for real-time PCR

Name	Sequence (5′→3′)	OAT, °C	GenBank ID
*RV*-QF	TCAGTTCGTCAGGAATATGC	53.5	AF317123
*RV*-QR	CTTGAAGGTGAGTAGTTGGT		
*GPR109a*-QF	ATGCTGGACCCTTTGGTGTAT	56.4	XM021072989
*GPR109a*-QR	GGCTTGTGCTGCGGTTATT		
*GRP78*-QF	TCGGCGATGCAGCCAAGAAC	59.8	XM001927795
*GRP78*-QR	CGGGTCATTCCATGTCCGGC		
*PERK*-QF	CTGCCACTTCAGCATCATTC	61.7	XM021086085
*PERK*-QR	TTCCATCCAGGTCACCACAT		
*IRE1*-QF	CGTCCTGGATCCAAAACT	54	XM005668695
*IRE1*-QR	GTCAGATAGCGCAGGGTCTC		
*ATF6*-QF	CCGAAGAGAAGAGCCATCTG	60.3	XM021089515
*ATF6*-QR	TCCTTTGATTTGCAGGGTTC		
*CHOP*-QF	CACTCTTGACCCTGCCTCTC	58.4	NM001144845
*CHOP*-QR	GACTGGAATCAGGCGAGTGT		
*Bcl-2*-QF	TGTGTGTGGAGAGCGTCAACC	62.5	XM021099593
*Bcl-2*-QR	CAGAGACAGCCAGGAGAAATCAA		
*Bax*-QF	CCACCAGCTCTGAGCAGATCA	61.3	XM003127290
*Bax*-QR	GCCGCCACTCGGAAAAA		
*Caspase9*-QF	GTCTGCCCACACCTAGTGAC	61.7	XM003127618
*Caspase9*-QR	AGGGGTCCCAGCCTCATTAT		
*Caspase3*-QF	TGGCGTGTCAGAAAATACCAGT	60.5	NM214131
*Caspase3*-QR	GATCCGTCCTTTGAATTTCGCC		
*β-actin*-QR	TCTGGCACCACACCTTCT	59.0	U07786
*β-actin*-QF	TGATCTGGGTCATCTTCTCAC		

### Western blotting

Protein was isolated from cells using cold lysis buffer containing a proteinase and phosphatase inhibitor cocktail (Beyotime, Shanghai, China). The protein concentrations in the supernatants were measured using a BCA protein quantification kit (Beyotime, Shanghai, China). Samples containing equal amounts of protein (20 μg) were separated by 10% SDS-polyacrylamide gel electrophoresis and then transferred to a polyvinyldifluoride membrane (Bio-Rad Co. USA). The membranes were blocked and then incubated overnight at 4 °C with primary antibody (PERK, Santa Cruz Biotechnology, catalogue no.sc-377,400; phospho-PERK (p-PERK), Abcam, catalogue no. ab192591; eIF2α, Cell Signaling, catalogue no.5324; phospho-eIF2α (p-eIF2α), Abcam, catalogue no. Ab32157; caspase9, Cell Signaling, catalogue no.9504; caspase3, Cell Signaling, catalogue no. D3R6Y; β-actin, Cell Signaling, catalogue no. D6A8), washed four times using TBST (5 min each time). Then, the membrane was incubated with the corresponding HRP-conjugated secondary antibody at 25 °C for 1 h, washed four times with TBST for 20 min, and visualized using ECL chemiluminescence kit (Beyotime, Shanghai, China). Finally, the Gel-Pro Analyzer was used to analyze protein densitometry. The relative expression levels of all protein were normalized to β-actin.

### Statistical analysis

All results are expressed as means ± SD. Data were analyzed using the statistical software SPSS 19.0 (SPSS Inc., Chicago, IL). All results were unpaired two-tailed Student’s T test and/or one-way analysis of variance (ANOVA). *P* < 0.05 and *P* < 0.01 were statistically significant (^*^) and markedly significant (^**^) respectively.

## Results

### RV induces ERS mediated apoptosis in IPEC-J2 cells

**Fig. 1:**
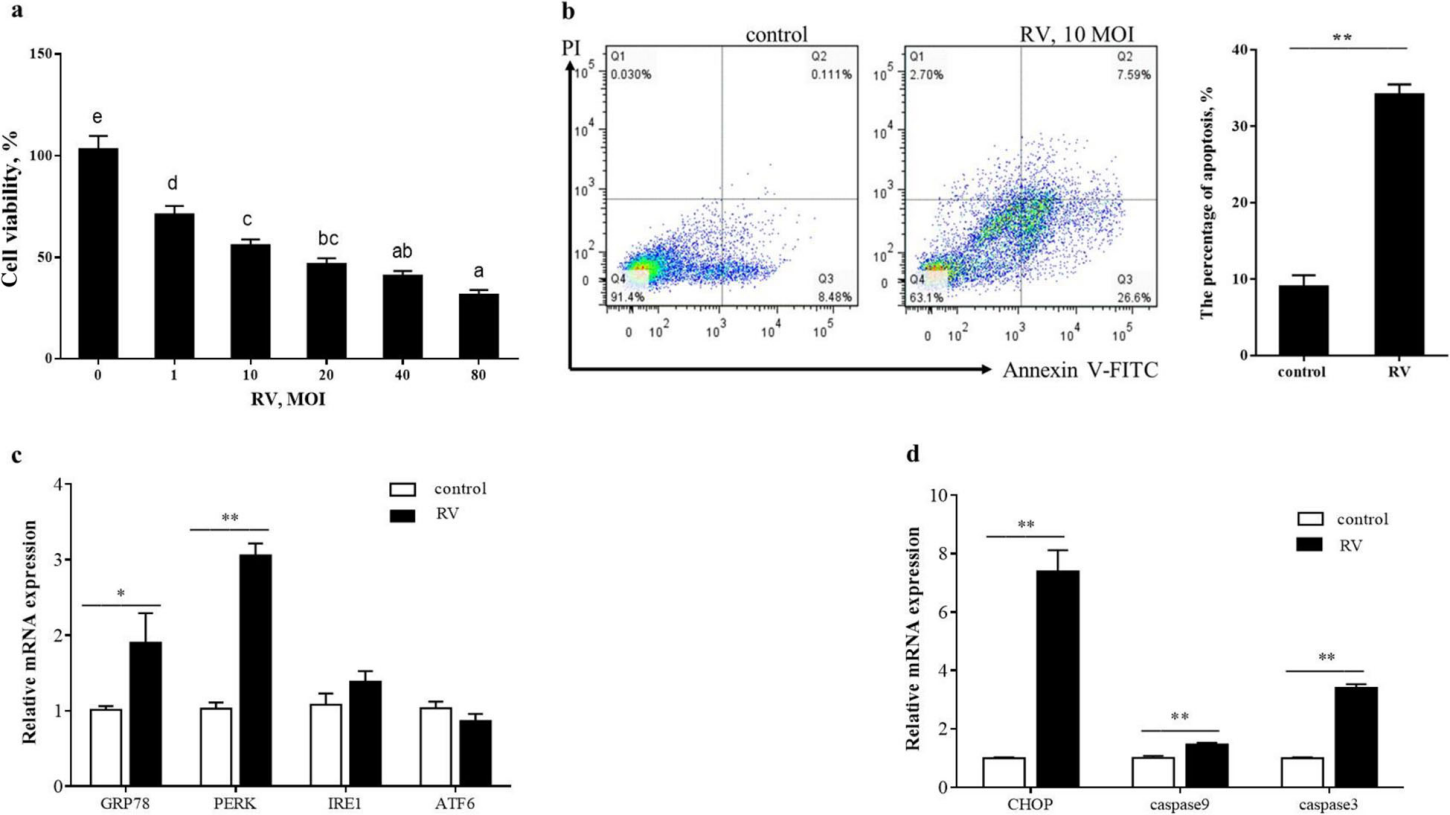
The effect of RV infection on cell viability and apoptosis in IPEC-J2 cells. **a** Cell viability was measured by CCK8 assay after RV infection for 24 h. b, Apoptotic populations of cells double stained with PI- and FITC-labeled Annexin V were measured by flow cytometry. **c** and **d** The effect of RV infection on the mRNA expressions of the ERS-mediated apoptosis related genes in IPEC-J2 cells. ^*^P < 0.05, ^**^P < 0.01, data are expressed as means ± S.D. from three independent experiments at least

**Fig. 2:**
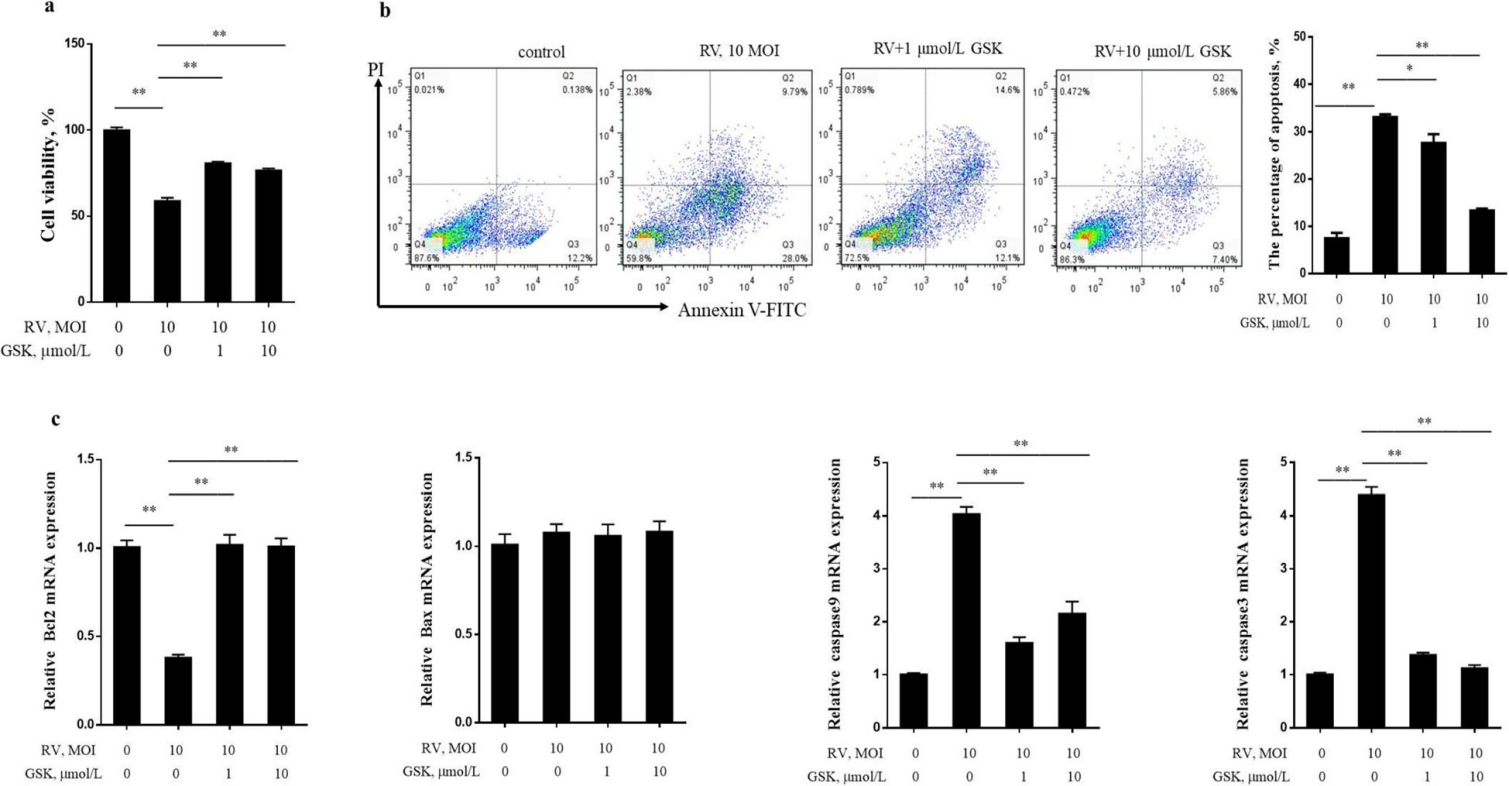
The effect of PERK inhibitor (GSK) on cell viability and apoptosis in IPEC-J2 cells. Cells were pre-treated with an apoptosis inhibitor (GSK, 1 or 10 μmol/L) for 24 h, followed by challenge with RV (10 MOI) for 1 h, and then incubated with DMEM/F12 for a further 24 h. **a** Cell viability was measured by CCK8 assay. **b** Apoptotic populations of cells double stained with PI- and FITC-labeled Annexin V were measured by flow cytometry. **c** The effect of RV infection on the mRNA expressions of apoptosis related genes in IPEC-J2 cells. ^*^P < 0.05, ^**^P < 0.01, data are expressed as means ± S.D. from three independent experiments at least

**Fig. 3:**
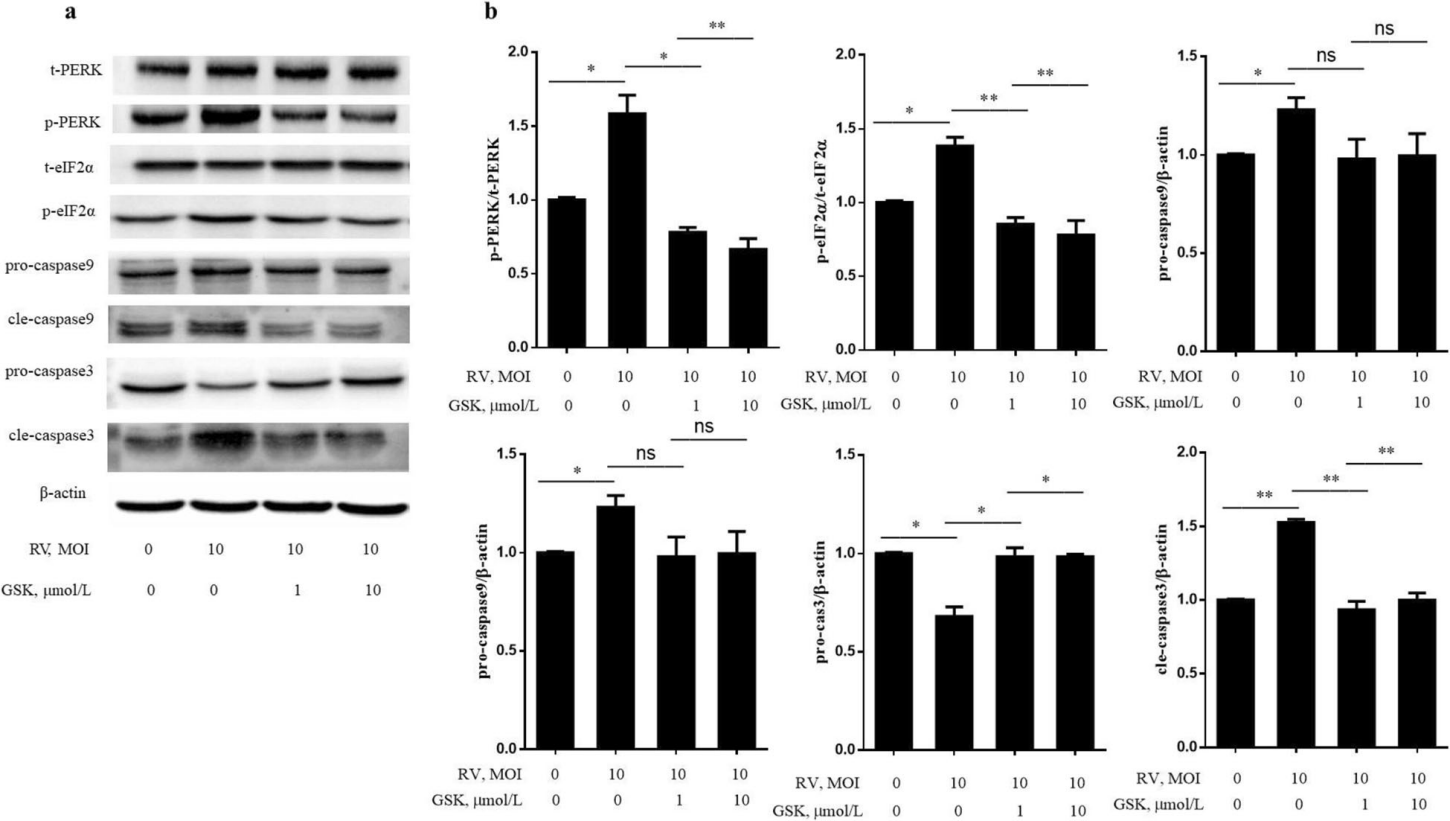
The PERK inhibitor (GSK) alleviated RV induced apoptosis by regulating PERK-eIF2α signaling pathway in IPEC-J2 cells. Cells were pre-treated with the PERK inhibitor (GSK, 1 or 10 μmol/L) for 24 h, followed by challenge with RV (10 MOI) for 1 h, and then incubated with DMEM/F12 for a further 24 h. **a** The t-PERK, p-PERK, t-eIF2α, p-eIF2α, pro-caspase9, cle-caspase9, pro-caspase3, and cle-caspase3 protein levels were determined by western blot. **b** Results were expressed as the ratio of p-PERK and t-PERK, p-eIF2α and t-eIF2α, pro-caspase9 and β-actin, cle-caspase9 and β-actin, pro-caspase3 and β-actin, and cle-caspase3 and β-actin protein levels. Equal loading was monitored with anti-β-actin antibody. ^*^P < 0.05, ^**^P < 0.01, data are expressed as means ± S.D. from three independent experiments at least

Initially, to define whether RV induce ERS mediated cell apoptosis, different assays were conducted in uninfected- and RV-infected IPEC-J2 cells at 24 h post-infection. As shown in the Fig. 1a and b, RV infection inhibited cell proliferation in a MOI-dependent mode. By using flow cytometry analysis, the percentage of apoptosis IPEC-J2 cells increased to 34.24% from a baseline of 9.08% after RV infection. In response to RV infection, an increase in the mRNA level of ERS marker *GRP78* was observed (Fig. 1c). As shown in Fig. 1c and d, the mRNA levels of *PERK*, *CHOP*, caspase9, and caspase3 were significantly increased in 24 h post infected cells. Inhibition of *PERK* by GSK potently promoted the proliferation (Fig. 2a) and down-regulated caspase9 and caspase3 mRNA expression (Fig. 2c) of RV-infected IPEC-J2 cells. The apoptosis rate in RV infected cells pretreated with GSK (10 μmol/L) was significantly decreased compared to that in RV infected cells (Fig. 2b). Western blot analysis showed that p-PERK/t-PERK, p-eIF2α/t-eIF2α, and cle-caspase9 and cle-caspase3 protein levels were significantly increased in 24 h post infected cells, while their protein levels were significantly decreased with *PERK* inhibitor treatment (Fig. 3a and b). These results collectively suggest that RV activates ERS and PERK-eIF2α signaling pathway, which maybe an important reason of RV-induced cell apoptosis.

### SB ameliorates RV induced ERS mediated apoptosis in IPEC-J2 cells

**Fig. 4:**
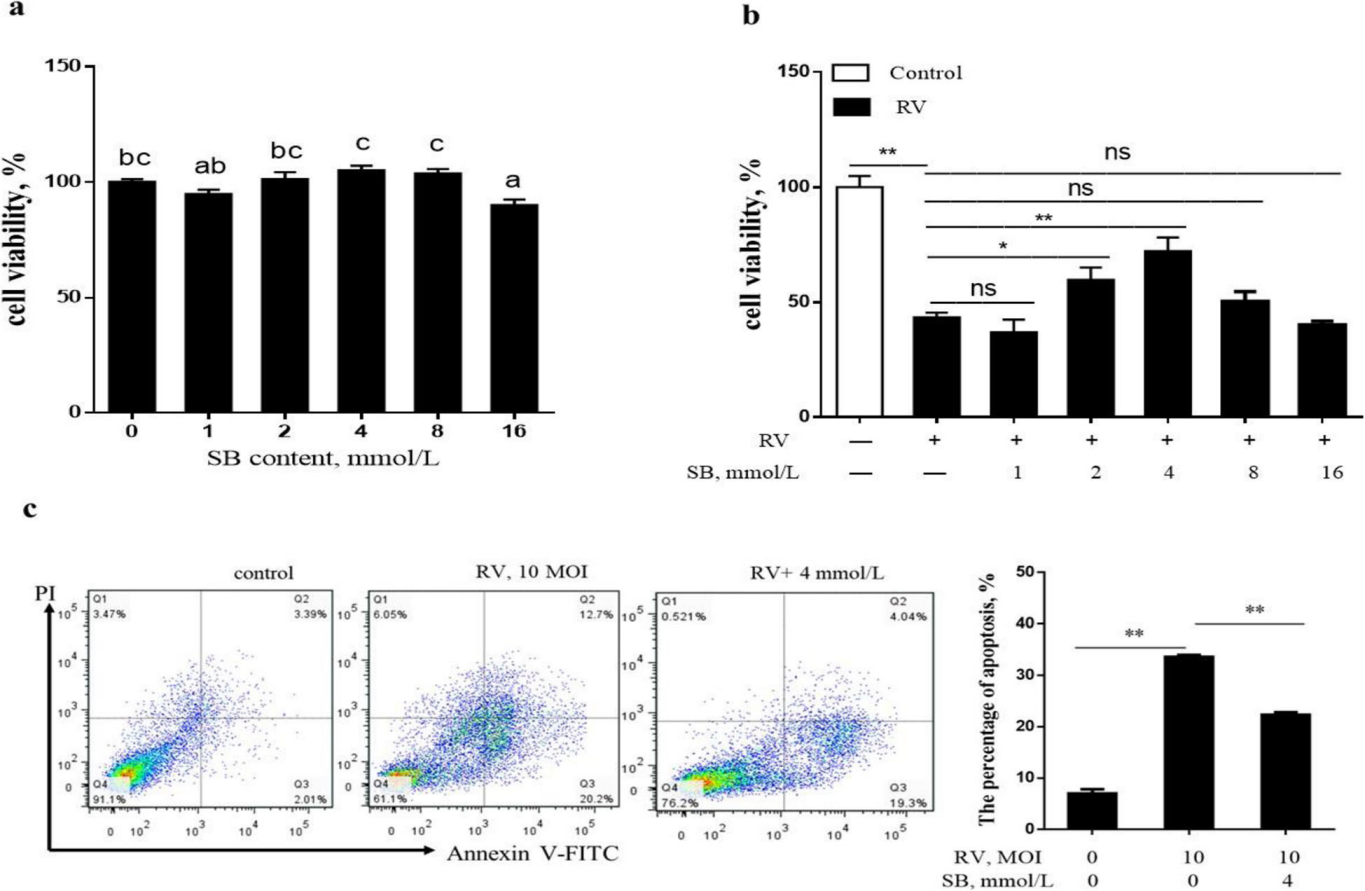
The effects of SB on proliferation and apoptosis in RV-infected IPEC-J2 cells. **a** The change of cell viability after treatment with SB (0, 1, 2, 4, 8, 16 mmol/L) for 24 h. **b** The IPEC-J2 cells were pretreated with SB (0, 1, 2, 4, 8, 16 mmol/L) for 24 h, followed by challenge with or without RV (10 MOI) for 1 h, and then incubated with DMEM/F12 for a further 24 h. Cell viability was detected by CCK-8 assay. **c** Apoptotic populations of cells double stained with PI- and FITC-labeled Annexin V were measured by flow cytometry. ^*^P < 0.05, ^**^P < 0.01, data are expressed as means ± S.D. from three independent experiments at least

**Fig. 5:**
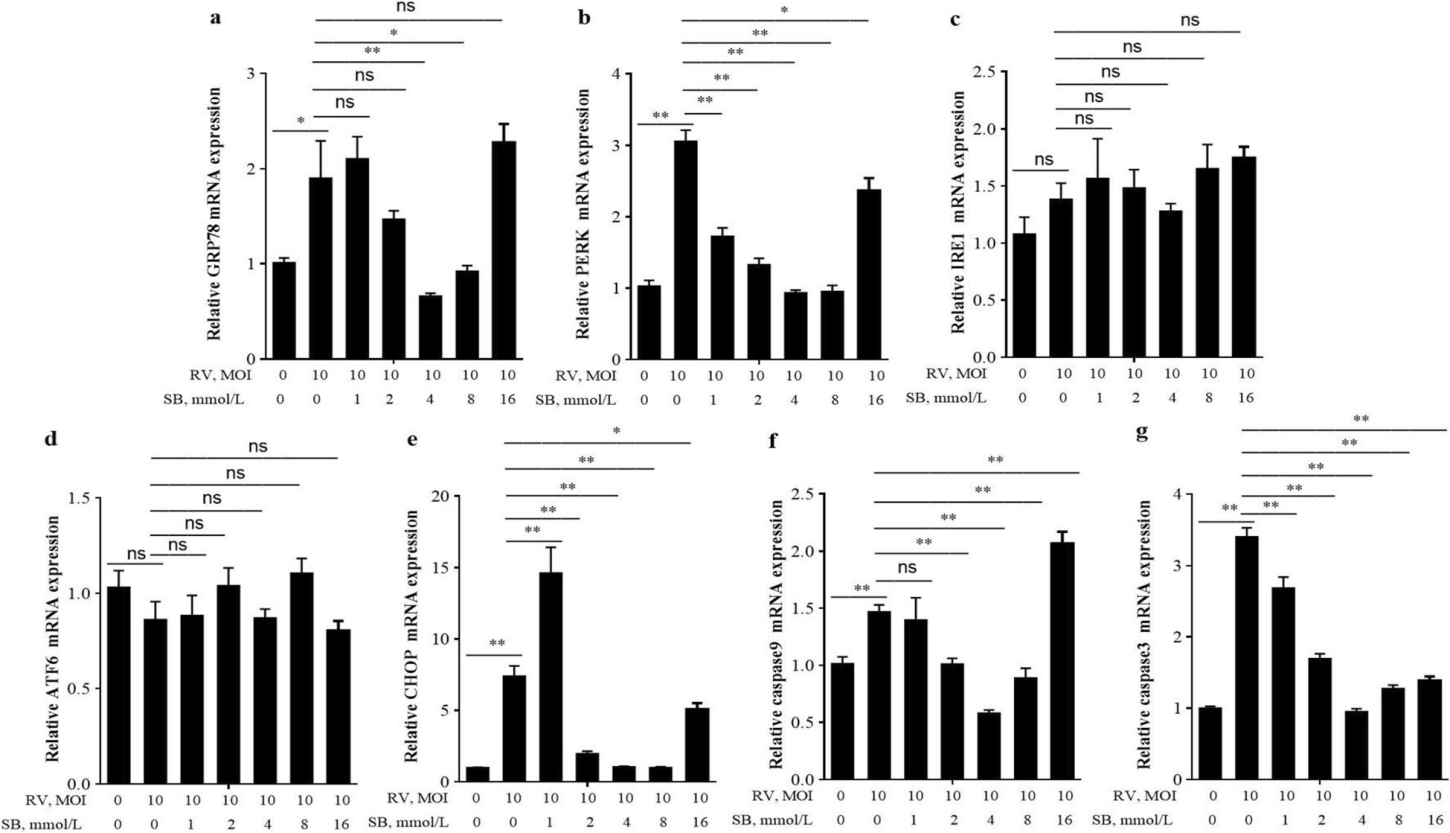
Effect of SB on ERS-mediated apoptosis related gene relative mRNA expressions in RV-infected IPEC-J2 cells. The IPEC-J2 cells were pretreated with SB (0, 1, 2, 4, 8, 16 mmol/L) for 24 h, followed by challenge with or without RV (10 MOI) for 1 h, and then incubated with DMEM/F12 for a further 24 h. **a**-**g** *GRP78*, *PERK*, *IRE1*, *ATF6*, *CHOP*, caspase9, and caspase3 mRNA expressions were measured by qRT-PCR. ^*^P < 0.05, ^**^P < 0.01, data are expressed as means ± S.D. from three independent experiments at least

To determine whether SB could exert a protective effect against RV induced cell apoptosis. This study first examined the effect of SB (0, 1, 2, 4, 8, and 16 mmol/L) on the IPEC-J2 cells viability. The SB had no cytotoxic effects up to the concentration of 8 mmol/L (Fig. 4a) and tended (2 and 4 mmol/L) to alleviate RV induced the decrease of the cell viability (Fig. 4b). Further the number of apoptotic cells after SB treatment was measured by using flow cytometry analysis. As expectedly, pretreatment of SB (4 mmol/L) significantly decreased the apoptosis in RV-infected IPEC-J2 cells (Fig. 4c). As shown in Fig. 5a, b, e, f, and g, pretreated with SB (4 and 8 mmol/L) cells showed a significant decrease in *GRP78*, *PERK*, *CHOP*, caspase9, and caspase3 mRNA expressions in RV infected IPEC-J2 cells. Together, these results suggested SB might ameliorate RV induced cell apoptosis via PERK-eIF2α signaling pathway in IPEC-J2 cells.

### SB ameliorates RV induced cell apoptosis via GPR109a in IPEC-J2 cells

**Fig. 6:**
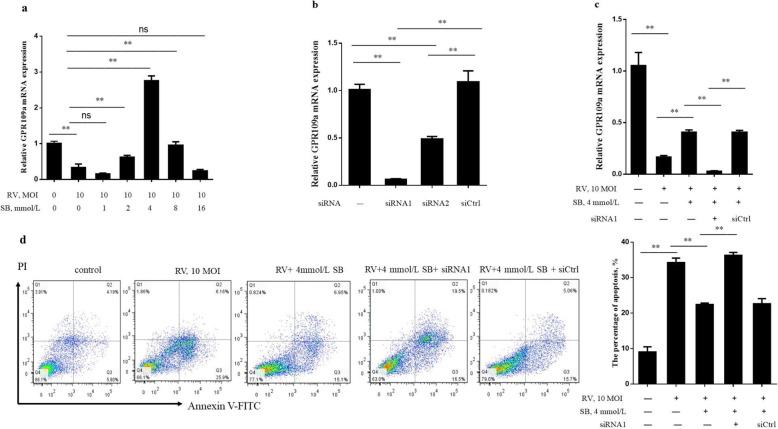
Protective effects of SB on RV induced apoptosis in IPEC-J2 cells. The IPEC-J2 cells were pretreated with SB (0, 1, 2, 4, 8, 16 mmol/L) for 24 h, followed by challenge with or without RV (10 MOI) for 1 h, and then incubated with SB (0, 1, 2, 4, 8, 16 mmol/L) for a further 24 h. **a** GPR109a mRNA expression was measured by qRT-PCR. **b** IPEC-J2 were transfected with constructed GPR109a small interfering RNAs (GPR109a siRNA1 and siRNA2) or its negative control (siCtrl). qRT-PCR was used to assess the mRNA expression of GPR109a. **c** IPEC-J2 were transfected with constructed GPR109a siRNA1. Then, cells were pretreated with SB (4 mmol/L) for 24 h, followed by challenge with or without RV (10 MOI) for 1 h, and then incubated with SB (4 mmol/L) for a further 24 h. **c** GPR109a mRNA expression was measured by qRT-PCR. **d** Apoptotic populations of cells double stained with PI- and FITC-labeled Annexin V were measured by flow cytometry. ^*^P < 0.05, ^**^P < 0.01, data are expressed as means ± S.D. from three independent experiments at least

**Fig. 7:**
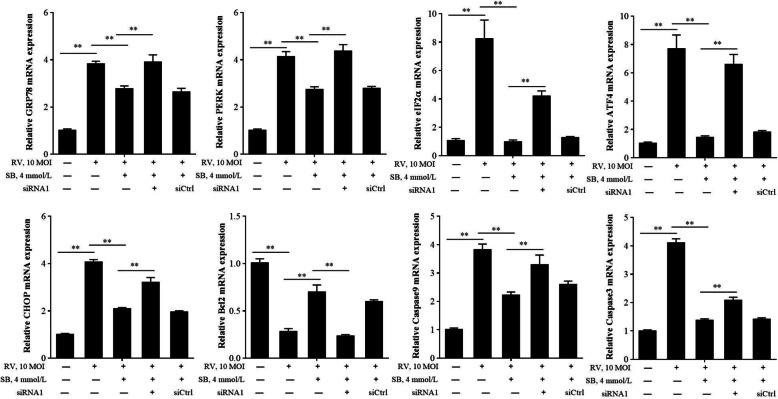
Effect of SB on ERS-mediated apoptosis related gene relative mRNA expressions in RV-infected IPEC-J2 cells after GPR109a knockdown. IPEC-J2 were transfected with constructed GPR109a siRNA1. Then, cells were pretreated with SB (4 mmol/L) for 24 h, followed by challenge with or without RV (10 MOI) for 1 h, and then incubated with SB (4 mmol/L) for a further 24 h. The *GRP78*, *PERK*, *eIF2α*, *ATF4*, *CHOP*, *Bcl2*, caspase9, and caspase3 mRNA expressions were measured by qRT-PCR

**Fig. 8:**
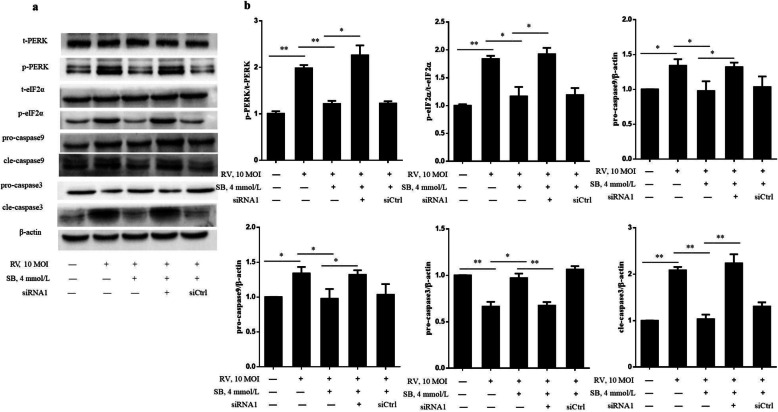
SB alleviated RV induced apoptosis by regulating PERK-eIF2α signaling pathway via GPR109a in IPEC-J2 cells. The IPEC-J2 were transfected with constructed GPR109a siRNA1. Then, cells were pretreated with SB (4 mmol/L) for 24 h, followed by challenge with or without RV (10 MOI) for 1 h, and then incubated with SB (4 mmol/L) for a further 24 h. **a** Protein expressions of t-PERK, p-PERK, t-eIF2α, p-eIF2α, pro-caspase9, cle-caspase9, pro-caspase3, and cle-caspase3 were determined by western blotting. **b** Results were expressed as the ratio of p-PERK and t-PERK, p-eIF2α and t-eIF2α, pro-caspase9 and β-actin, cle-caspase9 and β-actin, pro-caspase3 and β-actin, and cle-caspase3 and β-actin protein levels. Equal loading was monitored with anti-β-actin antibody. ^*^P < 0.05, ^**^P < 0.01, data are expressed as means ± S.D. from three independent experiments at least

To detect whether SB ameliorates RV induced cell apoptosis via GPR109a, this study first examined the effect of SB on GPR109a in RV infected IPEC-J2 cell. The SB (2, 4, and 8 mmol/L) significantly up-regulated GPR109a mRNA expression (Fig. 6a). Then, two small interfering RNAs against GPR109a (GPR109a siRNA1 and GPR109a siRNA2) were transfected into IPEC-J2 cell. Compared with siRNA control, the expression of GPR109a were significantly down-regulated by GPR109a siRNAs (Fig. 6b), and transfection efficiency of siRNA1 was more significant than siRNA2 (data not shown). After SB treatment, the mRNA expression of GPR109a was significantly decreased by GPR109a siRNA1 in RV infected IPEC-J2 cell (Fig. 6c). Therefore, GPR109a siRNA1 was chose in the following experiments. Flow cytometry assays indicated GPR109a siRNA1 significantly increased apoptosis rate in RV infected IPEC-J2 cell (Fig. 6d). Besides, GPR109a siRNA1 remarkably suppressed the effect of SB on *GRP78*, *PERK*, *eIF2α*, *ATF4*, *CHOP*, *Bcl2*, caspase9, and caspase3 mRNA expressions in RV infected IPEC-J2 (Fig. 7). Western blot results showed that SB strongly decreased protein levels of p-PERK, p-eIF2α, cle-caspase9, and cle-caspase3, but GPR109a siRNA1 attenuated this decrease in RV infected IPEC-J2 (Fig. 8a and b). In a word, these data revealed SB ameliorated RV induced cell apoptosis by regulating PERK-eIF2α signaling pathway via GPR109a.

## Discussion

As a major pathogenic factor, RV recently presents potential hazards to public health [40, 41]. Enterocytes are the first block of defence against the entry of pathogens in the gut lumen. Because of the significant physiologic and morphologic similarities to enterocytes *in vivo*, IPEC-J2 cell line has been widely applied to characterize the interactions of enterocytes with RV *in vitro* [42]. The RV predominantly invades epithelial cells in the proximal intestine thereby causing villous atrophy and crypt hyperplasia. Then it is also accompanied by deadly watery diarrhea, resulting in severe dehydration and death in human and animals [9]. Accumulating evidences demonstrated that RV infection leaded to apoptosis of intestinal epithelial cells, which is an important reason for RV induced diarrhea in animals [9, 16]. In this study, RV infection decreases viability of IPEC-J2 cells and increases the apoptosis rate. This result is consistent with a previous report in Caco-2 cells, which reported that RV infection leaded to the increase of DNA fragments and the loss of mitochondrial membrane potential [43]. Mao et al. also reported that RV infusion increased the apoptosis of the jejunal mucosal cells in piglet [16]. However, the present results indicated appropriate concentration of SB elevated the survival ratio and alleviated RV-induced cell apoptosis. These results indicate SB has an evident protective action against RV-induced IPEC-J2 cell apoptosis.

The PERK-eIF2α pathway is an important signaling pathway that regulates ERS mediated apoptosis after virus infection [26, 27]. The ER is the primary organelle for viral replication and maturation. Emerging evidences demonstrate that virus infection often disrupts the ER homeostasis and leads to activation of ERS [44–49]. The *GRP78* and *CHOP* are contemporary and novel biomarkers of ERS [50]. In this study, mRNA expressions of *GRP78* and *CHOP* were significantly increased in response to RV infection. These results suggested that RV infection might induce ERS. In normal, *PERK*, *ATF6*, and *IRE1* are bound by *GRP78*. When ERS is activated, three transmembrane proteins separate from *GRP78* that combines unfolded proteins. Subsequently, *PERK* and *IRE1* are activated by transautophorylation and *ATF6* is activated by proteolytic processing [51]. This study also found that RV infection significantly increased phosphorylation of *PERK* but not *IRE1* or *ATF6*. The *PERK* branch play a vital role in ERS related apoptosis. The activated *PERK* phosphorylates *eIF2α* on Ser51 site, inhibiting protein translation and synthesis. Subsequently, phosphorylated *eIF2α* selectively initiates the translation of *ATF4*, which is required in the apoptotic response to ERS [52]. In this study, p-PERK, p-eIF2α, cle-caspase9, and cle-caspase3 protein levels were significantly increased in RV infection cell, and caused a significant increase in cell apoptosis. To further demonstrate the direct involvement of PERK in RV-induced IPEC-J2 cell apoptosis, GSK was applied to verify the role of RV in ERS as a typical selective inhibitor of the PERK pathway. In this study, inhibition of *PERK* by GSK effectively reduced the expression of p-PERK and p-eIF2α, and RV-induced cell apoptosis. These results strongly suggested that the PERK-eIF2α pathway is critically involved in RV induced cell apoptosis.

The SB is a mineral form of short-chain fatty acid that plays essential roles in regulating cell apoptosis [35, 53, 54]. The previous study indicated that SB attenuated ERS induced islet β-cell apoptosis via inhibiting PERK-eIF2α signaling pathway in type 2 diabetic rats [36]. To further elucidate the potential mechanism of SB’s protective effect on RV-induced cell apoptosis in IPEC-J2, this study examined the levels of ERS- and apoptosis-related proteins. The present results showed protein levels of p-PERK, p-eIF2α, cle-caspase9, and cle-caspase3 were highly increased in response to RV infection. Moreover, pretreatment with SB effectively decreased p-PERK and p-eIF2α, cle-caspase9, and cle-caspase3 protein levels. Collectively, these results indicated that SB ameliorated RV-induced apoptosis through PERK-eIF2α signaling pathway. To our knowledge, this study is the first report demonstrating that SB protects IPEC-J2 against RV-induced apoptosis through inhibiting PERK-eIF2α signaling pathway.

The GPR109a is a G protein-coupled receptor for butyrate and expresses in intestinal epithelium [55], which have been explored as mediators of the biological effects of short-chain fatty acids [56]. Butyrate has attracted more attention that it not only plays an important role in anti-inflammatory and immune regulation, but also participates in the protection against intestinal cancer in a GPR109a-dependent manner [32, 57–59]. The present study found SB increased GPR109a mRNA expression in IPEC-J2 cells. The siRNA-mediated gene silencing of GPR109a blunts the anti-apoptosis effect of SB and blocks SB-mediated suppression of PERK-eIF2α signaling pathway, indicating that the protective role of SB might be related to the activation of GPR109a. This result was in good agreement with previous reports in piglet and mice. The SB ameliorates the 2, 4, 6-trinitrobenzene sulfonic acid-induced inflammatory response and disruption of epithelial integrity through activating GPR109a [32] and exerted its anti-diarrheal effect on weaned piglets by up-regulating the expression of colon tight junction protein in a GPR109a-dependent manner [60]. Taken together, these data indicate SB alleviates RV-induced apoptosis by regulating PERK-eIF2α signaling pathway via GPR109a.

## Conclusions

In conclusion, this study was the first to provide evidence that SB alleviated RV-induced apoptosis by regulating PERK-eIF2α signaling pathway via GPR109a. These results highlighted a novel mechanism of SB in regulation of RV-induced apoptosis in intestinal epithelial cells.

## Data Availability

The datasets produced and/or analyzed during the current study are available from the corresponding author on reasonable request.

## Abbreviations

RV: Rotavirus
ER: Endoplasmic reticulum
SB: Sodium butyrate
ERS: Endoplasmic reticulum stress
*PERK*: Protein kinase-like ER kinase
*eIF2α*: Eukaryotic initiation factor 2 alpha
*GRP78*: Glucose regulated protein 78
*CHOP*: C/EBP homologous protein
*ATF4*: Activating transcription factor 4
MOI: Multiplicity of infection
CCK8: Cell Counting Kit-8
FITC: Fluorescein isothiocynate
PI: Propidium iodide
